# Disparities in Intimate Partner Violence among Currently Married Women from Food Secure and Insecure Urban Households in South Ethiopia: A Community Based Comparative Cross-Sectional Study

**DOI:** 10.1155/2018/4738527

**Published:** 2018-09-20

**Authors:** Eshetu Andarge, Yohannes Shiferaw

**Affiliations:** ^1^Reproductive Health Unit, Department of Public Health, College of Medicine and Health Sciences, Arba Minch University, P.O. Box 21, Arba Minch, Ethiopia; ^2^Department of Public Health, Arba Minch College of Health Sciences, P.O. Box 155, Arba Minch, Ethiopia

## Abstract

Intimate partner violence (IPV) against women and food insecurity are an ever growing public health concerns in Ethiopia. However, the connection between the two is not yet well investigated. Therefore, this study compares IPV by household food security status and examines the association between IPV and food insecurity and among currently married women in childbearing age in Arba Minch town, South Ethiopia. A community based comparative cross-sectional study was conducted among 696 currently married women (15-49). A quantitative data was collected using a pretested and structured questionnaire on randomly selected women. Logistic regression was done using IBM SPSS version 20. Statistical significance was declared at p-value of less than 0.05 and odds ratio with its 95% confidence interval was used to show the degree of association. Lifetime and current IPV were 62.4% and 50%, respectively. Sixty-three (23.6%) and 285 (66.4%) women who experienced current IPV were from food secured and insecure households, respectively (P-value<0.001). The odds of experiencing current IPV were higher among women from food insecure households (AOR=6.59,95% CI(4.54,9.57)) and who were in the age range of 30-39 (AOR=2.16, 95% CI(1.34,3.56)) and it was lower among women with 10 and more years age difference with their husband (AOR=0.52, 95% CI (0.31,0.88)) and with high decision-making power (AOR=0.61, 95% CI (0.38.0.96)) than their reference groups. The prevalence of current IPV was significantly higher among women from food insecure households as compared to their counterparts. The odds of experiencing current IPV were higher among women from food insecure households. Policy makers and programme planners should consider improving urban women's household food security status in order to improve gender inequality and empower women. Multisectorial effort is needed to improve women education and other behavioral factors.

## 1. Introduction

Intimate partner violence (IPV), also known as spousal abuse or domestic violence, is defined as “behavior within an intimate relationship that causes physical, sexual, or psychological harm, including acts of physical aggression, sexual coercion, psychological abuse, and controlling behaviors”[1]. Worldwide, 40-70% of all female murder victims are killed by an intimate partner, with no country safe from the violence acts. Around the world, at least one in every three woman (5 times that of males) has been beaten, coerced into sex, or otherwise abused in her lifetime; the abuser being her own family in most cases. In the US, poorer women experience significantly more domestic violence than women from higher economies [2, 3]. According to the World Health Organization (WHO) multicounty study, more than 50% of women in four countries (Bangladesh, Ethiopia, Peru, and Tanzania) reported physical or sexual violence by intimate partners, 71% in rural South Ethiopia [4]. Studies from different parts of Africa also revealed consistent estimates from Zambia (about half), Uganda (46%), Tanzania (60%), Kenya (42%), and Nigeria (81%) [5]. IPV against women is common both in urban and in rural families of Ethiopia. About 88% of rural and 69% of urban women believe that their husbands have the right to beat them [6]. Like in the other parts of the world, for many Ethiopian women and girls, “home” is where they face a regime of terror and violence at the hands of somebody close to them, somebody they should be able to trust[7].

Intimate partner violence against women, taking forms of physical, sexual, mental harm, or suffering to women, has significant negative maternal and child health outcomes. When it occurs among pregnant women, it may end up in pregnancy loss, preterm labor, pregnancy complications, and hypertension, delivering low birth weight, physical injuries, and stress [4, 8, 9]. Resulting in child abuse, neglect with in the household, and depression of women, IPV against women has an impact on the growth and nutritional status of the children of the affected women [10]. The link between IPV and reproductive health issues is also a major concern in public health as IPV increases the risk of acquiring sexually transmitted infections including Human Immune Virus (HIV), risky sexual health behavior, and miscarriage [11, 12]. A study from the Spanish National Health Survey (2006) showed that the likelihood of coronary heart disease, chronic neck, and back pain was higher among women who reported IPV than among those who did not [13]. In a recent survey on violence against women (2015) in the same country, different forms of violence revealed varying degrees of physical and mental problems like anxiety, sadness because of feelings of worthlessness, wanting to cry for no reason, mood swings, irritability, insomnia, sleep disorders, permanent fatigue, and lack of sexual desire [14].

Intimate partner violence against women is major obstacle on progress to achieving development targets [9]. The 2030 agenda for sustainable development has set goals, the Sustainable Development Goals (SDGs) [15], which offer the opportunity to prevent all forms of violence. Goal 5 addresses gender equality and the empowerment of women and girls where one of the targets (Target 5.2) is set out to “eliminate all forms of violence against all women and girls in public and private spheres”. Thus, the current global development agenda has addressed all forms of violence against women and girls, as well as violence against children for the first time in its history [16].

Existing literatures suggest further research on the connections between poverty and IPV against women as it occurs in all social and economic classes [17] though women living in poverty are more likely to experience violence. Women are victims of IPV at a rate about 5 times that of males. In US, domestic violence is most prominent among women aged 16 to 24. Poorer women experience significantly more domestic violence than higher income women [2, 12].

In Ethiopia, domestic violence is a concern not only from a human rights perspective but also from an economic and health perspective. The country has put in place appropriate and effective legal and policy provisions to promote the rights of women and girls. The government of Ethiopia revised its family law in 2000, its criminal law, and constitution in 2005 in addition to ratifying many of the international and continental agreements that promote and protect women's rights, including the Convention on the Elimination of Discrimination against Women (CEDAW), and the Protocol to the African Charter on the Rights of Women in Africa. In line with this, the government has also put in place the requisite institutional mechanisms at federal and regional levels [18]. The current Growth and Transformational Plan (GTP II 2015) has, for the first time, included ending violence against women as a priority [19]. Depending on the severity of damage inflicted, legal penalties range from small fines to imprisonment for up to 10 to 15 years [20]. However, about a third (34%) of ever-married women aged 15-49 have experienced intimate partner violence. Physical and emotional violence were experienced by 24% each and sexual violence by 10% [18]. Domestic violence is illegal, but government enforcement of laws against rape and domestic violence was inconsistent. It was underreported due to cultural acceptance (societal norms), shame, fear, or a victim's ignorance of legal protections [20].

With 10% of the population in chronic food insecurity, Ethiopia takes one of the highest food insecurity conditions in the world [21]. It is not only a rural problem, urban food insecurity is also a mounting concern owing to fluctuating food prices and great dependency of urban households on food supplied by the market [22]. For illustration, 75% of households in Addis Ababa are food insecure [23]. Food insecurity situations, particularly among men with low socioeconomic status who cannot provide food for their families, may force them to be frustrated and hopeless which in turn could lead them to violence, particularly against their wives or partners [24]. Such situations could also reinforce discrimination of women and girls increasing the occurrence of domestic violence [25]. A recent study from California, USA, among randomly selected hetrosexual women of 18 years and above showed a strong positive association between food insecurity and IPV [26]. In Ethiopia, a study in Sidama Zone has also showed a similar association [27].

As evidences suggest, both food insecurity and IPV are common occurrences in Ethiopia. Even though there were studies in the country on IPV, majority of them focused on physical violence alone, while sexual and emotional abuse are also determinants of women and children's health. Moreover, estimates of IPV were also limited to the Northern and Western parts of the country and to the best of our knowledge only one study in Western Ethiopia, Agaro, a small town in Jimma Zone, was conducted entirely among urban women [23, 28–35]. The existence of such discrepancies among study findings on IPV indicates the importance of site-specific studies especially in urban parts of our country where studies are very scanty. Moreover, the connection between food insecurity and reproductive health rights in the context of IPV is not well documented in the country in general and in the study area in particular. Thus, this study is designed with a different outlook to comprehend the relationships between the two explicitly among currently married women in an urban setting of Arba Minch town, Southern Ethiopia.

## 2. Methods and Materials

### 2.1. Study Setting and Design

The study was conducted from March 10-20, 2017, in Arba Minch town. Arba Minch is one of the towns in Southern Nations, Nationalities and People's Regional (SNNPR) state which is a capital town of Gamo Goffa zone and the surrounding district, ”Arba Minch Zuria Woreda”. It is located 505 kms to the South of Addis Ababa, the capital city of Ethiopia. The town is one of the towns in the Great Rift Valley having a hot climate. Administratively, the town is divided into to four subcities. There is one general hospital, two health centers, and different levels of private health facilities. Women of reproductive age accounts for 23.30% (25,654) of the total population projected for the year 2009 EC which was 110,104 (male =54,832 and female =55,272) [36]. A community based comparative cross-sectional study was conducted among currently married women of childbearing age (15-49 years). The study compares intimate partner violence among the target women from food secure and insecure households in the town.

### 2.2. Sample Size and Sampling Procedure

The sample size for this study was determined using a formula for estimation of two population proportion using Epi info software version 7 with the assumptions of 95% confidence level, a 50% expected proportion of intimate partner violence in the last 12 months among food secure currently married women, 80% power, the ratio of food secure to food insecure households (r = 2), and odds ratio (OR)=2. The sample size calculated using these assumptions was 335. With consideration of a design effect of 2 for cluster sampling and a nonresponse rate of 10%, a total sample of 737 married women of childbearing age were included in the study.

Arba Minch town has 11 kebeles (the smallest administrative unit in the government structure of Ethiopia) [36]. The study was conducted in four kebeles among randomly selected women who were currently married and living with their husband. Women who were seriously sick or unable to give information to the data collectors and those who were not permanent residents (lived in the town for less than 6 months) were excluded from the study. Multistage cluster sampling was used to select the study subjects. Four kebeles (Chamo, Mehal Ketema, Woze, and Wuha Minch) were selected out of 11 kebeles by using simple random sampling (lottery method). Then, a frame of households with the target women was prepared for the four kebeles based on the current list of households with married women for the year 2009 EC (2016/2017 GC). The list was obtained from a secondary data from kebele administration based on recent reports of block leaders (coordinators of some households in the villages of a kebele). Hence, total number of currently married women was 1181, 1875, 2988, and 2277 for Chamo, Mehal Ketema, Woze, and Wuha Minch kebeles, respectively. The calculated sample size was proportionally allocated to each study kebele based on the total number of the selected households with currently married women they have. Ultimately, random sampling was employed using SPSS to identify the required number of respondents (Chamo=105, Mehal Ketema=166, Woze=264, and Wuha Minch=202) from the frame of households in each kebeles (S1 Figure). In a situation when a household had two or more eligible subjects, only one was selected by lottery method to control the potential intrahousehold correlation. At times when the woman was not present at her home during data collection, an attempt was made to get her two times after the first visit and if she was absent, a woman in the next order in the list was included and then, subsequent households were included according to the already predetermined order. A woman who refuses to participate in the interview was considered as nonrespondent.

### 2.3. Data Collection Method, Tools, and Procedure

A total of 8 data collectors were recruited for data collection. The data collectors were female students who completed high school education and residents of Arba Minch town. Two health officers were recruited for supervision of the data collection. The data collectors were given the list of women to be interviewed in their respective kebeles in advance on the date of data collection. During the data collection period, a health development army leader in each kebele guided the data collectors and supervisors so that they can easily access the houses of each sampled woman. A two-day long intensive training was given to the data collectors and supervisors on the data collection process. The details of the training focused on clarification of the problem, the significance of the study for public health practice, objectives, methodology with particular emphasis on how to conduct the survey and measurement, and every section of the instrument for data collection. They were thoroughly trained on the importance of interviewing women in a private place and referral of victims of violence with serious risk (women who had sign and symptoms of depression and or anxiety, having serious physical injury and suicidal and homicidal ideations). An interactive lecture followed by group discussion and role-play on the data collection procedures and measurement of variables and the elements of the questionnaire were conducted by the principal investigator and facilitators. A pretested, structured, interviewer-administered questionnaire was used. To assess intimate partner violence a questionnaire partly adapted from the standard WHO multicountry study questionnaire for assessing women's health and domestic violence [4] and for the household food insecurity and the Food Insecurity Experience Scale developed by FAO [37] validated for use in different social contexts [38, 39] were used. The questionnaire was first prepared in English and then translated to Amharic and to realize its validity, back translation to English language was made by an expert having skills in both languages.

### 2.4. Data Analysis

Data was entered in to Epi-data V3.1 and then exported to IBM SPSS statistics version 20 and was cleaned for inconsistencies and missing values and analyzed. Descriptive statistics using frequencies, percentages, mean, and standard deviations were used to describe findings. Cross-tabulation between each explanatory variable and the outcome variable was conducted and the fulfillment of assumptions of chi-square was ascertained. Bivariate analysis using logistic regression was done for the variables that fulfill the assumptions for chi-square and all explanatory variables that have association with the outcome variable at p-value of less than 0.25 were selected as candidates for multivariable analysis. Prior strong significance was also considered for selection of some variables for multivariable analysis. Multicollinearity between the candidate variables was checked. Then multivariable analysis was done to control for possible confounding variables. Model fitness was checked using Hosmer and Lemeshow goodness of fitness test. Presence of statistically significant association between explanatory variables and the outcome variable was declared at p- value < 0.05 and OR with its 95% CI was used to measure the degree of association between independent variables and the outcome variable.

### 2.5. Data Quality Management

The quality of data was assured by proper design and pretest of the questionnaire, proper training of the interviewers, and supervisors of the data collection procedures. Every day, 10% of the completed questionnaires were reviewed and checked for completeness and relevance by the supervisors and principal investigator and the necessary feedback was offered to data collectors in the next morning before the actual procedure. The data collection was thoroughly supervised by the principal investigator and supervisors. The questionnaire used in the survey was translated to the local language and back translated to English language to check for its original meaning.

The questionnaire was pretested on a kebele that is not included in the sampled cluster in the study area on the 33 respondents (5% of sample size). Findings were discussed among data collectors, supervisors, and the investigator in order to ensure better understanding to the data collection process. Based on the pretest, questions were revised and edited, and those found to be unclear or confusing were modified. Finally, a structured Amharic version questionnaire was used for data collection. Each woman was interviewed in a separate private place to avoid social desirability bias and protect the victims from further violence.

### 2.6. Definitions and Measurement

- Intimate partner violence among currently married women (15-49) was defined as women who experience any of physical, psychological, or sexual violence; it was coded as Yes = 1 (women who experience any of physical, psychological, or sexual violence) and No = 0 (women who do not experience any of the three forms of violence acts).
- Physical violence meant if the woman reports that she had been slapped or had something thrown at her; pushed or shoved; hit with a fist or something else that could hurt; kicked, dragged, or beaten up; choked or burnt; threatened with or had a weapon used against her.
- Sexual violence meant if the woman reports that she had been physically forced to have sexual intercourse; had sexual intercourse because she was afraid of what her partner might do; been forced to do something sexual she found degrading or humiliating.
- Psychological violence meant if the woman reports that she had been said or done something to humiliate her in front of others close to her; threaten to hurt, or harm her or insult her or make her feel bad about herself.
- Current intimate partner violence: women who reported that they experienced any of the three forms (physical, sexual, or psychological) of intimate partner violence acts 12 months preceding the survey time were considered as having current intimate partner violence.
- Life time intimate partner violence: women who reported that they experienced any of the three forms (physical, sexual, or psychological) of intimate partner violence acts any time in their marital life.
- The Food Insecurity Experience Scale consists of an eight item questionnaire defining and measuring food insecurity based on the perspective of the study women who experience it in the last 12 months. The questions are labeled from 1 to 8 from least insecurity to severest insecurity experiences (6 questions, 3 each indicating mild to moderate food insecurity and 2 questions indicating severe food insecurity conditions): worrying about how to procure food, compromising on quality and variety, reducing quantities, skipping meals, and experiencing hunger. The study women respond on their perspectives representing their household members. A food secure household experiences none of the food insecurity conditions (those who answer “no” to all the questions about food insecurity-related experiences). A food insecure household experiences at least one of the food insecurity conditions, mild to severe (those who answer “yes” to at least one of the food insecurity-related experiences) [37].
- Woreda refers to an administrative level corresponding to district in other parts of the world.
- Illiterate refers to those women who were not able to read and write.
- Intimate partner refers to current spouse (husband) in this study.
- Currently married women refers to those women who have been married and are not either divorced, widowed, or separated during data collection.
- The index for decision-making power was composed of four questions. The women were asked “who in her family usually has the final say on the following decisions”: (1) how to use the money earned by her or her partner, (2) healthcare for herself, (3) major household purchases, and (4) visits to her family or relatives. The possible responses for each item were respondent alone, respondent and husband/partner jointly, husband/partner alone, or someone else. For each items the response was scored as 2 if a woman made sole decision, 1 if she was involved with someone (husband/partner or someone else), and 0 otherwise; the sum of the scores were made to represent an overall index of a woman's decision-making power. The total score on decision-making power was 8. Hence, those women who scored four and above were categorized as having high decision-making power whereas those scored less than four were categorized as women with low decision-making power.
- A woman's partner (husband) was taken as a drug user when she reports that her husband was either a user of alcohol or khat (if she replies yes for either of the questions on her partner's alcohol consumption or khat chewing).

### 2.7. Ethics Approval and Consent to Participate

Ethical clearance was obtained from Arba Minch College of Health Sciences Ethical Review Committee. A formal letter of permission to conduct the study was obtained from Arba Minch town health office and each selected kebele. Written consent on willingness to participate in the study was obtained from the study subjects. As part of the consent process, the right of the participant to stop the interview when she was uncomfortable and information on confidentiality procedures was assured in advance. In order to assure the privacy and security of participants, the interview was conducted in a private setting and the information was kept confidential with the research team and the interviewed woman. Each interviewer was assigned out of her own kebele of residence. No names or identifiers were included on the questionnaire.

## 3. Result

### 3.1. Sociodemographic and Behavioral Characteristics of the Respondents by Their Food Security Status

A total of 696 currently married women participated in the study yielding a response rate of 94.4%. The mean age of the participants was 31±7. The mean age of their partner's was 37±11. More than two-thirds 443(63.6%) of the study women had less than 10 years difference in age from their partner. More than half 367(52.7%) of the study participants were orthodox religion followers. About two-thirds 414 (59.5%) of the study women were from Gamo ethnic group. Concerning women's educational status, more than two-thirds 471(67.6%) attended secondary education and above.

Majority 611(87.7%) of the respondents arranged their current marriage by their own love; 364(84.8%) and 244(93.1%) of them were from food insecure and food secured households, respectively. Regarding duration of marriage in completed years, nearly two-thirds 421(62.6%) of the study women were in their current marriage for less than 10 years; 264(63.8%) and 157(60.6%) of them were from food insecure and food secured households, respectively. A sizeable proportion of women 124 (17.9%) had low decision-making power of which 92(21.4%) and 32(12.2%) were from food insecure and food secured households, respectively. Three hundred and ten (45.0%) of the study women's partners use drugs (alcohol or khat) of which 204(47.6%) and 106(40.8%) were from food in secure and food secure households, respectively. Majority 623(90.2%) of the study participants have the habit of attending mass medias with 382 (89.0%) and 241 (92.0%) of them from food insecure and secured households, respectively. Four hundred seventy-six (76.9%) of the mass media attendants attended it daily. The remaining details are shown below (Table 1).

### 3.2. Life Time Intimate Partner Violence among Currently Married Women in Arba Minch Town, South Ethiopia

**Figure 1:**
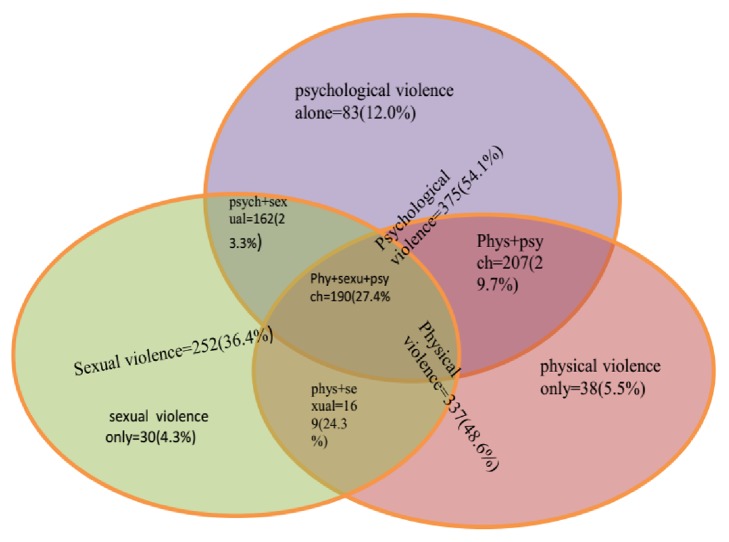
Venn diagram on lifetime experience of IPV. The figure illustrates overlaps between psychological, physical, and sexual violence and the isolated occurrences among currently married women aged 15-49 years in Arba Minch town, South Ethiopia, March 2017.

Out of the total women participated in the study, four hundred thirty-one (62.4%) of them experienced intimate partner violence at some point in their life of marriage. More than half (54.1%) of the study participants experienced psychological violence with an isolated psychological violence 83(12.0%). Physical and sexual violence were experienced by 337(48.6%) and 252(36.4%) of women, respectively. The isolated experience of physical and sexual life time violence was 38(5.5%) and 30(4.3%), respectively. The overlapping experiences of psychological, physical, and sexual violence were 190 (27.4%) (Figure 1).

### 3.3. Comparison of Current Intimate Partner Violence among Currently Married Women from Food Secured and Insecure Households in Arba Minch Town

Considering reports on psychological, physical or sexual violence, 348 (50%) of women experienced violence from their intimate partner 12 months before the study time; sixty-three (23.6%) and 285(66.4%) of those who experienced violence were from food secured and insecure households, respectively (chi-square (x^2^) test p-value<0.001). Two hundred eighty-three (40.7%) experienced psychological violence; 53(19.9%) and 230(53.6%) of them were from food secured and insecure households respectively (x^2^ test p-value<0.001). More than a third of women 252 (36.2%) experienced physical violence 12 months preceding the study time; 32(12%) and 220(51.3%) of the victims of physical violence were from food secured and insecure households, respectively (x^2^ test p-value<0.001). Nearly a third of women experienced sexual violence 207(29.7%). Out of these women, 28(10.5%) and 179(41.7%) were from food secured and insecure households respectively (x^2^ test p-value<0.001). The sole and overlapping occurrences are also illustrated (Table 2).

### 3.4. Factors Associated with Life Time Intimate Partner Violence among Currently Married Women of Childbearing Age in Arba Minch Town

A total of 14 variables (age of women, partner's age, occupation of woman and her partner, women's decision-making power, drug use, family size, average monthly income, educational status of women and her partner, marriage arranger, women's exposure to mass media, marital duration in years, and age difference between women and her partner) were analyzed in the bivariate logistic regression; 12 of which showed an association with lifetime intimate partner violence at a p-value of less than 0.25 (women's occupation and marital duration did not show such an association). The 12 candidate variables were fit to the multivariable logistic regression model after checking for multicollinearity. Hence, women's education, marriage arranger, decision-making status, and their partner's drug use were found to be factors associated with their lifetime experience of intimate partner violence.

Women with secondary education and higher had 0.55 times lower odds of experiencing intimate partner violence than those with no formal education (AOR=0.55(0.32, 0.97)). Women whose marriage was arranged without their personal choice (parents/guardians/by abduction) had 1.75 times higher odds of experiencing intimate partner violence than those who arranged their marriage by their own choice (love) (AOR=1.75(1.01,3.06)). Women with high decision-making power had 0.45 times lower odds of experiencing life time intimate partner violence than those with low decision-making power (AOR=0.45(0.28,0.73)). Women with drug using partners had 2.29 times higher odds of experiencing life time intimate partner violence than those with nonuser partners (AOR=2.29(1.64,3.23)) (Table 3).

### 3.5. Factors Associated with Current Intimate Partner Violence among Currently Married Women of Childbearing Age in Arba Minch Town

A total of 15 variables (age of women, partner's age, occupation of woman and her partner, women's decision-making power, household food security status of women, drug use, family size, average monthly income, educational status of women and her partner, marriage arranger, women's exposure to mass media, marital duration in years, and age difference between women and her partner) were analyzed in bivariate logistic regression; seven of them (partner's age, women's age difference with partner, women's educational status, women's and partner's occupation, women's household food security status, and women's decision-making power) showed an association with current intimate partner violence at p-value of less than 0.25. These candidate variables were fit to the multivariable logistic regression model after checking for multicollinearity. Women's age, age difference with her husband, decision-making power, and food security status were found to be factors associated with their current experience of intimate partner violence.

Women with age of 30-39 years had 2.16 times higher odds of experiencing current intimate partner violence than those in the age of below of 30 years (AOR=2.16(1.34,3.56)). A higher age difference between couples showed lesser odds of experiencing intimate partner violence in this study. Women with 10 years and above age difference with their husband had 0.52 times lower odds of experiencing current intimate partner violence than those with less than 10 years difference (AOR=0.52(0.31,0.88)). Similarly, a higher decision-making power by women showed a lesser odds of women's experience of current intimate partner violence. Women with high decision-making power had 0.61 times lower odds of experiencing current intimate partner violence than those with low decision-making power (AOR=0.61(0.38.0.96)). The odds of experiencing current intimate partner violence were 6.59 times higher among women from food in secure households than those from in secure households (AOR=6.59(4.54,9.57)) (Table 3).

## 4. Discussion

This study examined whether food insecurity is independently associated with intimate partner violence and compared its independent relation with different forms of intimate partner violence. The overall prevalence of IPVAW in this study is relatively lower than studies elsewhere in Ethiopia [4, 27, 31, 33, 40]. For instance, the findings from Butajira (71% and 54%) and East Wollega (76.5% and 72.5%) women experienced lifetime and current IPVAW, respectively. The difference could be owing to the fact that in this study the respondents were entirely from an urban area. This might be explained as gender relations in urban regions are more distant from traditional patterns and greater presence of women's movements and support services [41]. Moreover, urban women might be more educated, have exposure to mass medias, and able to protect their legal rights as compared to rural women. It could also be due to differences in the study participants wherever married/cohabited women were the study participants in most of the above studies that include women already divorced, separated, or cohabited and one of the likely causes for these events could be violence against women.

However, lifetime prevalence of intimate partner violence against women (IPVAW) in this study (62.4%) was higher than a study in Agaro town (51.8%), Gondar Zuria district (50.8%), and the recent national demographic and health survey report for urban women (29.4%) [29, 30, 42]. Cultural differences may explain the discrepancy between the aforementioned studies and the present study and this implies that intimate partner/domestic violence is still highly prevalent in varying degrees across the country calling for further efforts by the concerned bodies. In this study, lifetime and current (last 12 months) psychological, physical, and sexual intimate partner violence were 54.1%, 48.6%, and 36.4% and 41.0%, 36.5%, and 30.0%, respectively. This shows that majority of the types of violence were experienced 12 months before the survey time. This was consistent with findings from studies conducted in East Wollega and Agaro town [29, 33] which implies that violence against women has become a norm in Ethiopia because of the deep-rooted traditions which support patriarchal family. Across time, there is minimal reduction in intimate partner violence despite the concerted efforts by the government and other stakeholders to reduce gender inequality and this has implications for further efforts. It could also be explained by the fact that women might forget or they may intentionally obscure previous violence acts if there is improvements in their partner's behavior in recent times because of fear of further aggravation or oppressions.

Marital rape is one of the gender based violence acts by an intimate partner which is committing nonconsensual sex in a consensual marriage [33]. The 2005 amended Criminal Code of Ethiopia [43] ignored forced sexual acts in marital relationship as a crime. Forced sexual acts in intimate relationship was abundant in this study. It accounts for 36.4% during women's lifetime and 30.0% in the past 12 months though it was lower than 58.7%, 51.0% and 46%, 33% lifetime and past 12 months violence experiences in the study from East Wollega and Butajira, Ethiopia, respectively [33, 40]. The difference could be attributable to urban-rural differences in the occurrence of the violence act as partners might be more educated in the urban setting despite the fact that urban women are less tolerant and more likely to report on sexual violence than rural women [31]. This implies that forced sexual acts with in matrimony were accepted within the community and would likely be underreported due to absence of legal recognition and protection.

The overlap of physical and psychological violence was the commonest occurrence as compared to the other two joint occurrences. This is in line with studies from Ethiopia, Nicaragua, and South Africa [33, 44, 45]. This can be explained by the fact that psychological violence like threatening and psychological attack accompany physical violence at much of the time [33, 46]. Moreover, the WHO multicountry study on VAW states that the most acts of physical violence reflect a pattern of abuse rather than an isolated incident [4]. The isolated occurrences of all the three types of violence were relatively minimal in this study. However, there is an overlapping of the three forms of violence in 27.4% and 20.8% of women in their lifetime and last 12 months, respectively. The finding was lower than findings from East Wollega and Butajira (56.9% and 42%), respectively, presenting a slight progress across time, though it is still much higher figure in the country's efforts to achieve gender equality in lights of the SDGs [19].

In the present study, currently married women from food insecure households had a significantly higher experience of overall and specific forms of psychological, physical, and sexual or both forms of current intimate partner violence (P-value<0.001). The odds of experiencing intimate partner violence in the last 12 months were much higher among women from food insecure households than those from food secured households. This is consistent with a previous studies from California in the united States of America, Ethiopia, Bangladesh, USA, and Atlanta [26, 27, 47, 48]. At times when men lack the resources associated with their assumed dominant role of a male breadwinner, they are more likely to express their frustration through violence. The condition of food insecurity may operate through different mechanisms such as instilling fear in men and promote hopelessness, stress, frustration, and a sense of inadequacy among men for having failed to live up to their culturally expected role as providers [27, 41, 49]. On the other side, it could also be owing to the stress created by the lack of food that might prompt women to demand and blame their partners for not performing the breadwinner role that could in turn provoke conflict and end up in violence acts.

In the study, women in the age range of 30-39 had higher odds of experiencing current intimate partner violence than those women under 30. This could be because of the bulk of women with child rearing experiences as housewives are concentrated in this middle age and younger women were more likely to be educated about women's right and employed. Women with 10 years and above age difference with their husband had lower odds of experiencing current intimate partner violence than those with less than 10 years difference. A previous study from Bangladesh also supports this [47]. This implies that older husbands were less likely to be abusive as they were matured and well prepared for marriage life. Moreover, the more close ages between women and men give more power and agency to women to demand their rights to choice in decisions in relationship and could lead to violence because men may not agree with women's agency. Similarly, women with high decision-making power were having lower odds of experiencing current and lifetime intimate partner violence than those with low decision-making power. The finding is consistent with other studies in Ethiopia, Zimbabwe, and Bangladesh [31, 47, 49, 50]. This implies that women having involvement in the decision of their household matters were likely to be educated and accepted by their partners and hence they could be in a better position to have a joint decision which in turn decreases the likelihood of experiencing violence.

Higher-level education resulted in lower odds of experiencing lifetime intimate partner violence in this study. This is consistent with other studies in Ethiopia [33, 50]. As education improves one's ability of rational thinking and understanding of human right issues advocated on mass medias, women with a higher education could be in a better position to protect their rights. This could also be because of the fact that women with a higher education were more likely to be employed and have a higher autonomy in decision-making which in turn lowers their experience of intimate partner violence. Women whose marriage was arranged by somebody else (parents/guardians/relatives/abduction) had higher odds of experiencing lifetime intimate partner violence than those who arranged their marriage by their own choice (love). The finding is consistent with findings of a previous study from East Wollega [33]. This could be for the fact that marriage initiated by the couple's joint decision is more likely to have a peaceful relationship as the couples could agree on most of household matters since they love each other. This contradicts with the traditional norm of initiation of marriage between couples by the will of family members that might particularly limit the right of women to choose their partner of marriage. This might lead to conflicts as there could be mismatch in age, behavior, and others.

Women with drug using partners had higher odds of experiencing lifetime intimate partner violence than those with nonuser partners. This is consistent with findings from population based surveys from Ethiopia, Nicaragua, and South Africa [27, 30–33, 44, 46]. Drug use (alcohol or khat) by partners was also a significant factor in reviews from Africa and Ethiopia [50, 51]. This could be because of the fact that drugs operate as a situational factor by clouding judgment, reducing inhibitions, and impairing an individual's ability to interpret cues and as a result increases the likelihood of violence.

This community based study, the first of its kind among urban women in the country, involved large sample size of 737 that reduces selection bias. However, the study has few limitations to consider. It has limitations inherent to cross-sectional studies; temporality cannot be established, resulting in the fact that the study evaluates associations rather than cause-and-effect relationships between the variables. Furthermore, owing to logistic reasons, census was not conducted to have the exact list of currently married women in the selected kebeles in the town. Because of the sensitive nature of both food insecurity and intimate partner violence, social desirability bias could be introduced and there will be either under reporting or over reporting. To reduce social desirability bias and to protect victims of violence from retaliation by their partners, data was collected in a private setting where no one else was able to hear the interview. Therefore, the findings of this study should be interpreted with consideration of these limitations. Longitudinal studies that take into account seasonal variations in household food security status of women need to be conducted to further validate the findings of this study.

## 5. Conclusion

The magnitude of intimate partner violence was high in this study. Nearly two-thirds and about half of the study women experienced at least one of the three forms of IPV during their life of marriage and in the past year preceding the survey time, respectively. The prevalence of current IPV was significantly higher among women from food insecure households as compared to their counterparts. Although a significant difference in IPV was observed between food secure and insecure households, the prevalence was high in the whole sample. The odds of experiencing current IPV were higher among women from food insecure households and age between 30 and 39 than their counterparts. Women with high decision-making power had lower odds of experiencing current and lifetime IPV. Similarly, women with an age difference of 10 or more years with their partner had lower odds of experiencing current IPV than those with less than 10 years difference. The odds of experiencing life time IPV violence were higher among women whose marriage was arranged by parents/guardians/abduction and with drug using partners. However, it was lower for those with secondary and higher-level education. The findings principally indicate that women's household food conditions affect their experiences of violence by their intimate partner through the pathway of challenging men's status of being breadwinners and providers to their family. The importance of education and empowerment of girls and women and behavioral factors like drug use by the partner have been also supported by this study.

Policy makers at federal and regional levels should be committed in improving urban women's household food security status in their efforts to improve gender inequality and end preventable maternal mortality. Moreover, scaling up women's autonomy through improvements in women's decision-making power in common household issues should also be their focus areas to reduce IPV. Education of girls and women should be strengthened through the concerted efforts of families and the education sector. The concerned bodies should promote interventions targeting behavioral and social factors affecting IPVAW. Moreover, extensive and longitudinal research is needed to validate the current findings.

## Figures and Tables

**Table 1:** Socio-demographic and behavioral characteristics on some variables among currently married women from food secure and insecure households in Arba Minch town, March 2017.

**Variables (N=696)**		**Total**	**Food security status**	
			Food insecure	Food secure
		Num(%)	Num (%)	Num (%)
**Partner's educational status**	No formal education	126(18.1)	84(19.6)	42(15.7)
	Primary level education	401(57.6)	236(55)	165(61.8)
	Secondary and above education	169(24.3)	109(25.4)	60(22.5)

**Women's occupation**	Housewife	308(44.3)	207(48.3)	101(37.8)
	Others^a^	388(55.7)	222(51.7)	166(62.2)

**Partner's occupation**	Government employee	310(44.5)	188(43.8)	122(45.7)
	Others^b^	386(55.5)	241(56.2)	145(54.3)

**Family size**	<5	552(79.3)	325(75.8)	227(85.0)
	5 and above	144(20.7)	104(24.2)	40(15)

**Average monthly income**	>=1317 ETB	547(78.6)	331(77.2)	216(80.6)
	< 1317 ETB	149(21.4)	98(22.8)	51(19.1)

**Partner drinks alcohol**	yes	264(37.9)	173(40.3)	91(34.1)
	no	432(62.1)	256(59.7)	176(65.9)

**Partner chews khat**	Yes	160(23)	108(25.2)	52(19.5)
	No	536(77.0)	321(74.8)	215(80.5)

**Mass media exposure**	yes	626(89.9)	382(89)	244(91.4)
	no	70(10.1)	47(11.0)	23(8.6)

^a^= employed, merchants, daily laborers, student, ^b^= merchants, farmer, carpenters, guards, etc.

**Table 2:** Comparison of current intimate partner violence among food secure and insecure currently married women in Arba Minch town, March 2017.

IPV last 12 months(N=696)	Category	Total	Food security status		p-value
		Num(%)	Food Insecure	Food Secure	
			Num(%)	Num(%)	
Psychological violence	No	413(59.3)	199(46.4)	214(80.1)	0.001
	Yes	283(40.7)	230(53.6)	53(19.9)	

Physical violence	No	444(63.8)	209(48.7)	235(88.0)	0.001
	Yes	252(36.2)	220(51.3)	32(12.0)	

Sexual violence	No	489(70.3)	250(58.3)	239(89.5)	0.001
	Yes	207(29.7)	179(41.7)	28(10.5)	

Intimate partner violence	No	348(50.0)	144(33.6)	204(76.4)	0.001
	Yes	348(50.0)	285(66.4)	63(23.6)	

Both psychological and physical violence	No	489(70.3)	248(57.8)	241(90.3)	0.001
	Yes	207(29.7)	181(42.2)	26(9.7)	

Both psychological and sexual violence	No	534(76.7)	290(67.6)	244(91.4)	0.001
	Yes	162(23.3)	139(32.4)	23(8.6)	

Both physical and sexual violence	No	527(75.7)	275(64.1)	252(94.4)	0.001
	Yes	169(24.3)	154(35.9)	15(5.60)	

Psychological, physical and sexual violence	No	552(79.3)	299(69.7)	253(94.80)	0.001
	Yes	144(20.7)	130(30.3)	14(5.2)	

Isolated psychological violence	No	638(91.7)	389(90.7)	249(93.3)	0.231
	Yes	58(8.3)	40(9.3)	18(6.7)	

Isolated physical violence	No	676(97.1)	414(96.5)	262(98.1)	0.212
	Yes	20(2.9)	15(3.5)	5(1.9)	

Isolated sexual violence	No	676(97.1)	413(96.3)	263(98.5)	
	Yes	20(2.9)	16(3.7)	4(1.5)	

**Table 3:** Factors associated with lifetime and current intimate partner violence among currently married women in Arba Minch town, March 2017.

**Variables (N=696)**		**IPV ever**		**COR (95**%** CI)**	**AOR (95**%** C.I)**
		yes	no		
		Num (%)	Num (%)		
Women's education	No formal education	91(21.1)	37(14.1)	1	1
	Primary level education	60(13.9)	33(12.6)	0.72(0.41,1.27)	0.66(0.36,1.23)
	Secondary education and above	280(65.0)	192(73.3)	0.58(0.38,0.89)	0.55(0.32,0.97)^b^

Marriage arrangement	Women's own choice (love)	366(84.9)	245(92.5)	1	1
	Others^a^	65(15.1)	20(7.5)	2.17(1.28,3.68)	1.75(1.01,3.06)^b^

Decision making	Low decision making	93(21.6)	33(12.5)	1	1
	High decision making	338(78.4)	232(87.5)	0.52(0.34,0.79)	0.45(0.28,0.73)^b^

Drug use by partner	Not user	207(48)	176(66.4)	1	1
	User	224(52)	89(33.6)	2.14(1.56,2.94)	2.29(1.64,3.23)^b^

Variables(N=696)		Current IPV			

Age of women	Less than 30	199(57.2)	234(67.2)	**1**	**1**
	30-39	104(29.9)	67(19.3)	1.01(0.58,1.74)	2.16(1.34,3.56)^b^
	40 and above	45(12.9)	47(13.5)	0.57(0.38,0.85)	1.21(0.64,2.29)

Difference in age	Less than 10 years	276(79.3)	240(69.0)	1	1
	10 or more years	72(20.7)	108(31.0)	0.58(0.41,0.82)	0.52(0.31,0.88)^b^

Decision making	Low decision making	81(23.3)	45(12.9)	1	1
	High decision making	267(76.7)	303(87.1)	0.49(0.33,0.73)	0.61(0.38.0.96)^b^

Household food insecurity	Food secure	63(18.1)	204(58.6)	1	1
	Food insecure	285(81.9)	144(41.4)	6.41(4.53,9.06)	6.59(4.54,9.57)^b^

^a^= Parents/guardians/relatives/marriage by abduction ^b^Statistically significant association at p-value < 0.05.

## Data Availability

The data used to support the findings of this study are available from the corresponding author upon request.

## Abbreviations

GTP: Growth and Transformation Plan
IPVAW: Intimate partner violence against women
IPV: Intimate partner violence
SRS: Simple random sampling
SPSS: Statistical package for social sciences
PPS: Probability proportion to size
WHO: World Health Organization.

## Supplementary Materials

Supplementary Materials S1 Figure: schematic presentation of the sampling procedure.
