# Supplementary material for: Disparities in Intimate Partner Violence among Currently Married Women from Food Secure and Insecure Urban Households in South Ethiopia: A Community Based Comparative Cross-Sectional Study
Source: Biomed Res Int. 2018 Sep 20;2018:4738527. doi: 10.1155/2018/4738527 (PMC6171209; doi:10.1155/2018/4738527)
Supplement: Supplementary Materials — S1 Figure: schematic presentation of the sampling procedure. [file 4738527.f1.docx]

Arba Minch town, 11 kebeles

SRS(lottery Method)

Mehal Ketema,WRA=1933,CMW=1875

Woze, WRA=3178,CMW=2988

Wuha Minch,WRA=2396,CMW=2277

Chamo WRA=1262,

CMW=1181

1875/8321=22.5% of 737=166

2988/8321=35.9% of 737=264

2277/8321=27.4% of 737=202

1181/8321=14.2%of 737=105

Key:

CMW=Currently married Women

SRS=simple random sampling (lottery Method)

WRA=Women of reproductive age
